# Comparative study on the antituberculous effect and mechanism of the traditional Chinese medicines NiuBeiXiaoHe extract and JieHeWan

**DOI:** 10.1186/s40779-021-00324-5

**Published:** 2021-06-02

**Authors:** Li-Yao Duan, Yan Liang, Wen-Ping Gong, Yong Xue, Jie Mi, Jie Wang, Lan Wang, Zai-Xing Jia, Hong Lei, Yu-Mei Liang, Jun Liu, Yue Zheng, Xue-Qiong Wu

**Affiliations:** 1 Tuberculosis Prevention and Control Key Laboratory, Beijing Key Laboratory of New Techniques of Tuberculosis Diagnosis and Treatment, Tuberculosis Research Institute, the 8th Medical Center, Chinese PLA General Hospital, Beijing, 100091 China; 2 HeBei North University, Zhangjiakou, 075000 China; 3 Clinical Laboratory, the 8th Medical Center, Chinese PLA General Hospital, Beijing, 100091 China; 4 Pathology Department, the 8th Medical Center, Chinese PLA General Hospital, Beijing, 100091 China; 5 Guangdong Qifang Pharmaceutical Co., Ltd, Guangzhou, 510075 China

**Keywords:** Traditional Chinese medicine, Tuberculosis, NiuBeiXiaoHe extract, JieHeWan, Therapeutic effect

## Abstract

**Background:**

The traditional Chinese medicine NiuBeiXiaoHe (NBXH) extract and Chinese medicine preparation JieHeWan (JHW) exhibit anti-tuberculosis effects. The anti- tuberculosis effect of NBXH was compared with that of JHW to elucidate the mechanism of action of NBXH.

**Methods:**

BALB/c mice aged 6-8 weeks were randomly divided into a normal control group, Tuberculosis (TB) model group, JHW treatment group, and NBXH treatment group. After 3 and 13 weeks of treatment, the therapeutic effect in each group was evaluated by comparing lung histopathology, lung and liver colony counts, the number of spots representing effector T cells secreting IFN-γ in an ELISPOT, and the levels of Th1, Th2, and Th17 cytokines, which were measured by a cytometric bead array (CBA). Mouse RNA samples were subjected to transcriptome sequencing.

**Results:**

After 13 weeks of treatment, the mean histopathological lesion area of the NBXH group was significantly smaller than that of the TB model group (*P* < 0.05). Compared with those in the TB model group, the lung colony counts in the JHW and NBXH groups were significantly decreased (*P* < 0.05), and the IL-2 and IL-4 levels in the NBXH group were significantly increased (*P* < 0.05). NBXH partly restored significant changes in gene expression caused by *Mycobacterium tuberculosis* (*M. tuberculosis*) infection. According to GO and KEGG analyses, the changes in biological process (BP), cell composition (CC) and molecular function (MF) terms and in signaling pathways caused by NBXH and JHW treatment were not completely consistent, but they were mainly related to the immune response and inflammatory response in the mouse TB model.

**Conclusions:**

NBXH had therapeutic effects similar to those of JHW in improving lung histopathology, reducing lung colony counts, and regulating the levels of cytokines. NBXH restored significant changes in gene expression and repaired cell damage caused by *M. tuberculosis* infection by regulating immune-related pathways, which clarified the mechanism of action of NBXH.

**Supplementary Information:**

The online version contains supplementary material available at 10.1186/s40779-021-00324-5.

## Background

Tuberculosis (TB) is a major social and public health problem worldwide. In 2019, there were 10 million new TB cases, and 1.2 million people died from TB worldwide [1]. In recent years, the rates of multidrug-resistant and extensively drug-resistant TB have been increasing. Anti-TB chemotherapeutic drugs have relatively high toxicity and side effects. The significant advantage of traditional Chinese medicine treatments is that they have a comprehensive therapeutic effect with “multicomponent, multitarget, and multisystem” features. Adjuvant treatment of multidrug-resistant tuberculosis (MDR-TB) with traditional Chinese medicine can effectively alleviate the symptoms of TB, promote the repair of TB damage, and treat or prevent adverse reactions to chemotherapeutic drugs. In China, there are rich resources for Chinese herbal medicine, and the treatment of TB with traditional Chinese medicine has a long history, which has accumulated rich experience and developed many mature prescriptions and traditional Chinese medicine preparations, such as JieHeWan (JHW), BaiHeGuJinTang, and KangLaoWan*.*

The traditional Chinese medicine prescription NiuBeiXiaoHe (NBXH) has been used in the clinic for 16 years. It is composed of 6 traditional Chinese medicines: Bulbus Fritillariae Cirrhosae, Rhizoma Bletillae, Herba Houttuyniae, Radix Platycodonis, Fructus Arctii, and glutinous rice. Among these components, Bulbus Fritillariae Cirrhosae and Rhizoma Bletillae are used as the monarch herbs. Herba Houttuyniae, Radix Platycodonis, and Fructus Arctii are used as the minister herbs, and glutinous rice is used as the adjuvant and guide herb. Clinical research has shown that NBXH containing these 6 traditional Chinese medicines with reasonable compatibility had the effects of nourishing Yin, moistening the lungs, relieving cough, resolving phlegm, clearing heat, detoxification, and softening hard modes, which could eliminate TB symptoms, such as low fever, emaciation, cough, chest pain, and hemoptysis [2–4]*.* A previous study by Wang XM found that NBXH had a certain therapeutic effect on a mouse TB model [5]. Li GM [6] found that NBXH might play an anti-TB role by inhibiting the energy metabolism, protein synthesis, and antioxidative stress of *M. tuberculosis*, weakening the viability of *M. tuberculosis* in macrophages and affecting latent infection. Ling YB et al. [7] found that NBXH has an immunomodulatory effect through proteomics, this effect may down-regulate the JNK signaling pathway and inhibit the inflammatory response. Chen D [8] found that NBXH has no significant effects on the nervous system, immune function, or liver and kidney functions of normal mice and do not cause an acute toxicity reaction.

The Chinese medicine preparation JHW is made of 16 traditional Chinese medicines including tortoise shell processing with vinegar, oyster, turtle shell processing with vinegar, Rehmannia, prepared Rehmannia, amethyst (calcined), asparagus cochinchinensis, honey-fried Radix Stemonae, keel, *Glehnia littoralis*, donkey hide gelatin, Radix Ophiopogonis, cooked rhubarb, Rhizoma Bletillae, Bulbus Fritillariae Cirrhosae, and beeswax. JHW has the effects of nourishing Yin, moistening the lungs, nourishing the kidneys, strengthening body resistance, protecting against TB and killing *M. tuberculosis*. The results reported by Zhang JG et al. [9] showed that JHW combined with anti-TB chemotherapeutic drugs could improve the immune function of the body, enhance the sensitivity to chemotherapeutic drugs, and alleviate the side effects of chemotherapeutic drugs.

In this study, the traditional Chinese medicine preparation JHW, which is used in the clinic, was used as a positive control to compare the anti-TB effects of NBXH and JHW for the first time in vivo, to study the effect of NBXH on multiple cytokines and gene expression profiles in a mouse infection model, to clarify the anti-TB mechanism of NBXH, to find a therapeutic effect evaluation index applicable to traditional Chinese medicine, and to provide a reliable experimental basis for the development of a new anti-TB traditional Chinese medicine preparation.

## Methods

### Traditional Chinese medicines

NBXH was prepared by Xi’an XinTong Pharmaceutical Research Company, Ltd. (Xi’an, China). JHW was produced by Anhui ZhiFeiLongKeMa Biopharmaceutical Company, Ltd. (Anhui, China).

### Mice

Specific pathogen-free female BALB/c mice, aged 6-8 weeks, 17–21 g, were obtained from the Vital River Laboratory Animal Technology Company, Ltd. (Beijing, China). They were maintained under barrier conditions in an animal room in the 8th Medical Center of the Chinese PLA General Hospital, Beijing, China. The experiments involving animals were approved and conducted by the Animal Ethical Committee of the 8th Medical Center of the Chinese PLA General Hospital, and mouse care met the standards of the Experimental Animal Regulation Ordinances defined by the China National Science and Technology Commission.

### *M. tuberculosis* strain

The standard *M. tuberculosis* strain H37Rv was purchased from the Chinese Academy for Food and Drug Control (Beijing, China). Colony forming units (CFUs) were used to determine amounts of viable bacteria by plating serial dilutions on Lowenstein-Jensen medium.

### Establishment of a mouse TB model and experimental grouping

BALB/c mice aged 6–8 weeks were injected intravenously via the tail vein with 3.1 × 10^5^ CFUs of *M. tuberculosis*. At 3 days after infection, 3 mice were randomly selected and euthanized, and their lungs and livers were taken for bacterial colony counting and pathological examination of lung tissue to observe whether the mouse TB model was successfully established. In addition, the other mice were randomly grouped. In the TB model group, 20 mice were gavaged daily with 0.5 mL of distilled water. In the JHW group, 20 mice were treated with 300 mg of JHW per kilogram of body weight (6 mg/0.5 ml/day). In the NBXH group, 20 mice were treated with 200 mg of NBXH per kilogram of body weight (4 mg/0.5 ml/day). In the normal control group, 20 mice were not infected with *M. tuberculosis* and were kept in an animal laboratory.

### Lung histopathological examination

The right lung of mice was fixed with 10% formalin buffer and embedded in paraffin. Sections were prepared by the paraffin-embedded tissues, stained with hematoxylin and eosin, and then analyzed by a registered pathologist.

### Bacterial counts

The left lung and upper liver of mice were homogenized in saline. Tissue suspensions were serially diluted 10-fold. In addition, 0.1 ml of each dilution was plated in duplicate on Lowenstein-Jensen plates and incubated at 37 °C for 4 weeks. *M. tuberculosis* colonies were enumerated on each plate, and the results are expressed as CFUs per organ.

### Enzyme-linked immunospot assay (ELISPOT)

A splenocyte suspension (3 × 10^6^/ml) and an ELISPOT plate were prepared in advance. The suspension was plated at 100 μl/well. One well contained 50 μl of complete cell culture medium, one well contained 50 μl of 60 μg/ml PHA, and three wells contained 50 μl of 60 μg/ml recombinant CE protein. The ELISPOT plate was incubated in a 37 °C cell incubator containing 5% CO_2_ for 24 h.

### Cytometric bead array (CBA)

A splenocyte suspension (3 × 10^6^/ml) was seeded in 48-well cell culture plates at 200 μl per well. Then, 200 μl of complete cell culture medium, 100 μl 60 μg/mL PHA, or 100 μl 60 μg/ml recombinant CE protein was added to each well. After incubation in a CO_2_ incubator at 37 °C for 48 h, the culture supernatant was collected. The levels of cytokines in the splenocyte culture supernatant were detected according to Th1/Th2/Th17 cytokine CBA kit (BD Company, America) instructions.

### PBMC isolation and total RNA extraction

Blood samples were collected from mice in an ethylenediaminetetraacetic acid dipotassium (EDTA) anticoagulant tube, and peripheral blood mononuclear cells (PBMCs) were isolated with the Mouse PBMC Isolation Kit (Haoyang Biological Products Technology, Tianjin, China). Total RNA was extracted from PBMCs using TRIzol Reagent (Life Biotechnology, Shanghai, China) according to the manufacturer’s instructions. The total RNA concentration and purity were determined using a NanoDrop 2000 (Thermo Fisher Scientific, DE, USA). RNA integrity was assessed using the RNA Nano 6000 Assay Kit for an Agilent Bioanalyzer 2100 system (Agilent Technologies, CA, USA).

### Transcriptome sequencing

Mouse RNA samples were subjected to RNA-Seq library preparation and transcriptome sequencing at Beijing Biomarker Biotechnology Co., Ltd. (Beijing, China). The sequencing libraries were generated using the Illumina NovaSeq platform (NEB, USA). Briefly, mRNA was enriched from total RNA using oligo (dT) magnetic beads. The mRNA was fragmented using divalent cations under an elevated temperature in fragmentation buffer. Using the mRNA as a template, the first cDNA strand was synthesized using random hexamer primers, and then the second cDNA strand was synthesized with buffer, dNTPs, RNase H, and DNA polymerase I. The generated double-stranded cDNA was purified with AMPure XP beads. Then, the purified double-stranded cDNA was subjected to end repair and poly(A) tail addition, and sequencing adaptors were ligated to the fragments. Fragments (240 bp) were purified with AMPure XP beads. The cDNA library was enriched by PCR. Finally, library quality assessment and quantification were performed on an Agilent Bioanalyzer 2100 system by Q-PCR, followed by sequencing on an Illumina NovaSeq platform.

### Data analyses

The significantly differentially expressed (DE) genes between the TB model group and normal control group and between the JHW or NBXH group and the TB model group were identified by volcano plots and hierarchical clustering. DE gene analysis between two groups was performed using the DESeq R package (1.10.1). Gene ontology (GO) enrichment analysis and Kyoto Encyclopedia of Genes and Genomes (KEGG) pathway analysis of the DE genes were implemented with the clusterProfiler R package.

### Statistical analyses

Statistical analysis of data was carried out using SAS (version 9.1, SAS Institute Inc., Cary, NC, USA). All the data are expressed as the mean ± standard deviation (SD). The significance of differences among groups was evaluated by one-way analysis of variance (ANOVA). A *P*-value less than 0.05 indicated statistical significance. Gene expression profiling of mice was analyzed by assessing fold changes. The threshold used to screen up- or down-regulated genes was |Fold Change| > 1 and *P-*value < 0.05.

## Results

### Histopathological changes

**Fig. 1:**
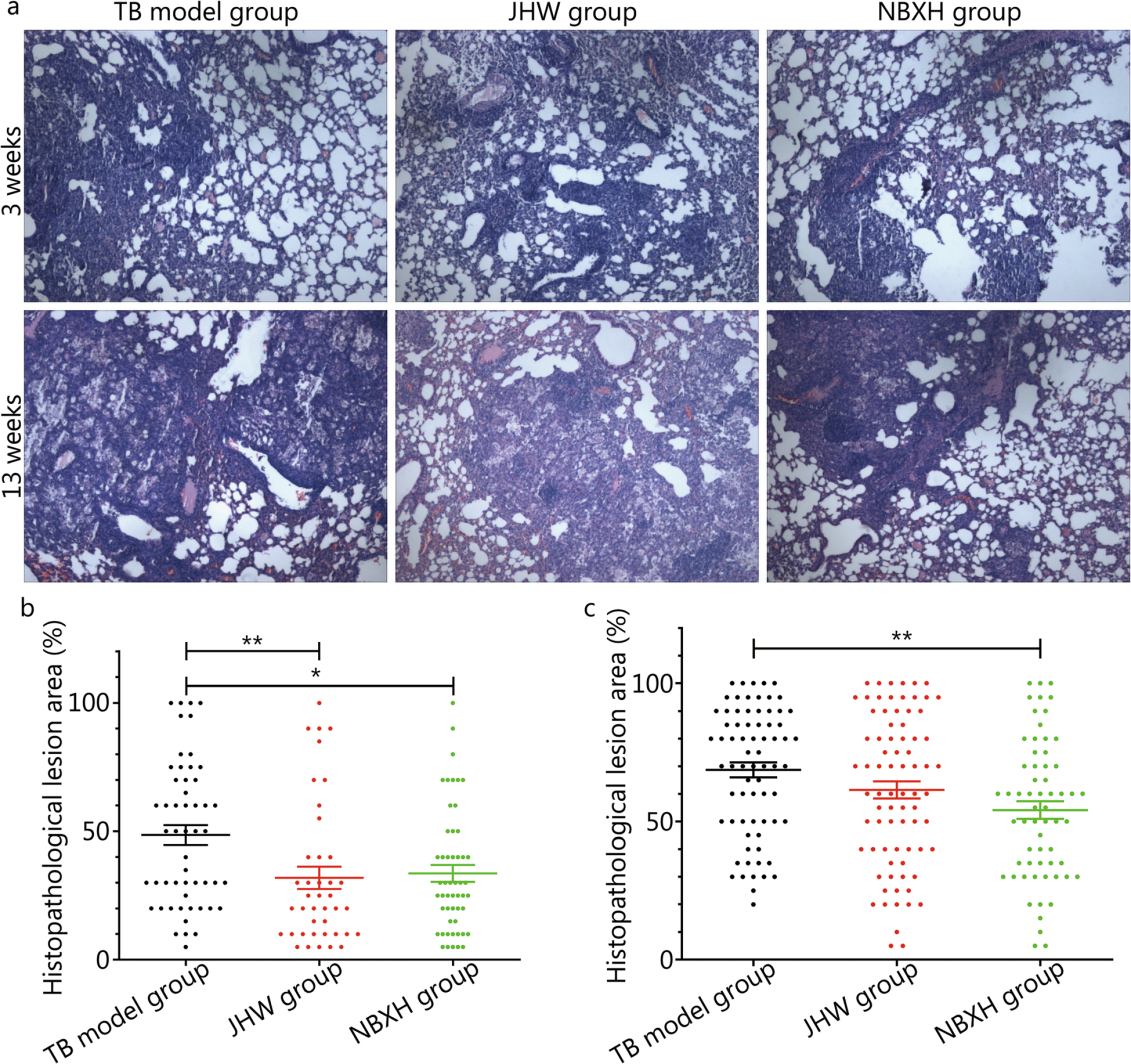
Representative photomicrographs and pathological lesion area of lung tissue in each group (HE stain, 40×) . **a** Lung tissues were obtained from tuberculosis (TB) model mice at 3 weeks and 13 weeks of treatment with distilled water, JieHeWan (JHW), or NiuBeiXiaoHe (NBXH), respectively. **b** Pathological lesion area of lung tissue after treatment for 3 weeks. **c** Pathological lesion area of lung tissue after treatment for 13 weeks. ^*^P < 0.05, ^**^P < 0.01

On the third day after infection, pathological examination of lung tissues from mice showed that the alveolar septum was congested, thickened, and infiltrated with inflammatory cells. After 3 weeks of treatment, lung tissue sections from mice in each group showed extensive pathological changes, destruction of the alveolar structure, congestion and thickening of the alveolar septum with lymphocyte infiltration, and multiple tuberculous nodules formed by lymphocyte aggregation (Fig. 1a). The pathological damage in the TB model group was more serious, with an average pathological damage area of more than 48.58%. Compared with that in the TB model group, the pathological damage in the JHW group and NBXH group was significantly reduced (*P* < 0.05), additionally, the pathological area of lung tissue in both groups was decreased (31.95 and 33.65%, respectively) (Fig. 1b). After 13 weeks of treatment, mouse lung tissue from each group showed severe pathological changes, including severe destruction of the alveolar structure and thickening and congestion of the alveolar septum, accompanied by a large amount of lymphocyte infiltration, and foam-like cells and lymphocytes aggregated to form tubercle nodules (Fig. 1a). The pathological damage in the TB model group was severe and extensive, and the average pathological lesion area was 68.68%. The average pathological lesion area in the JHW group (61.41%) was not significantly different from that in the TB model group (*P* > 0.05), that in the NBXH group (54.11%) was significantly smaller than that in the TB model group (*P* < 0.05, Fig. 1c).

### Bacterial counts in the lungs and liver

**Fig. 2:**
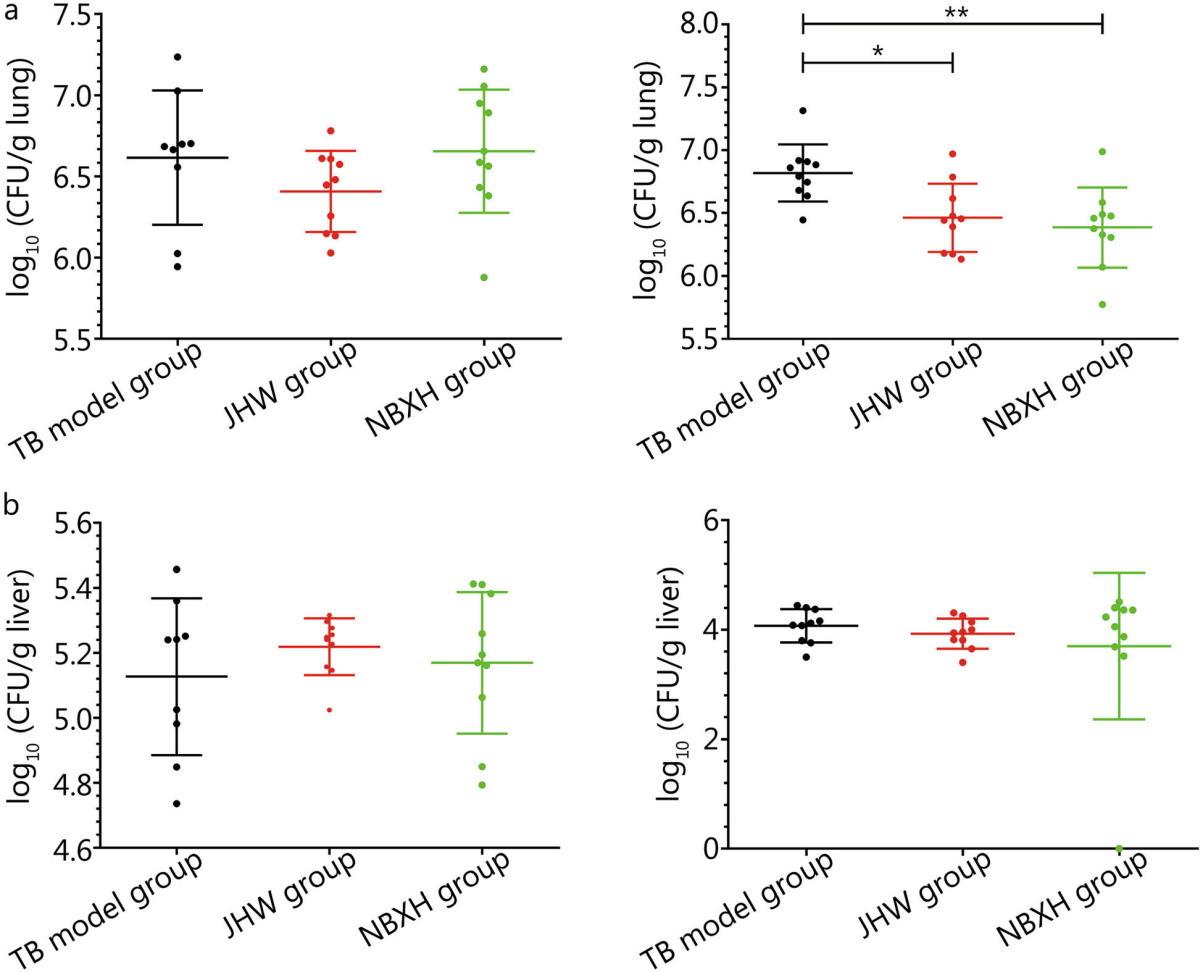
Numbers of live bacteria after treatment for 3 weeks (left) and after treatment for 13 weeks (right) in mice lungs (**a**) and livers (**b**). ^*^P < 0.05, ^**^P < 0.01

The lung colony counts of the TB model group at 3 days after infection were log_10_ (3.16 ± 0.22), and the liver colony counts were log_10_ (4.30 ± 0.29). After 3 weeks of treatment, compared with the TB model group, the JHW group and the NBXH group showed no significant differences in lung or liver colony counts (*P* > 0.05) (Fig. 2 left). After 13 weeks of treatment, the lung colony counts in the JHW group and the NBXH group were significantly lower than those in the TB model group (*P* < 0.05), and the lung colony counts in the NBXH group were lower than those in the JHW group (Fig. 2a right). Compared with the TB model group, the JHW group and the NBXH group showed no significant differences in liver colony counts (*P* > 0.05), but the liver colony counts in the NBXH group were relatively low (Fig. 2b right).

### Effector T cells secreting IFN-γ

**Fig. 3:**
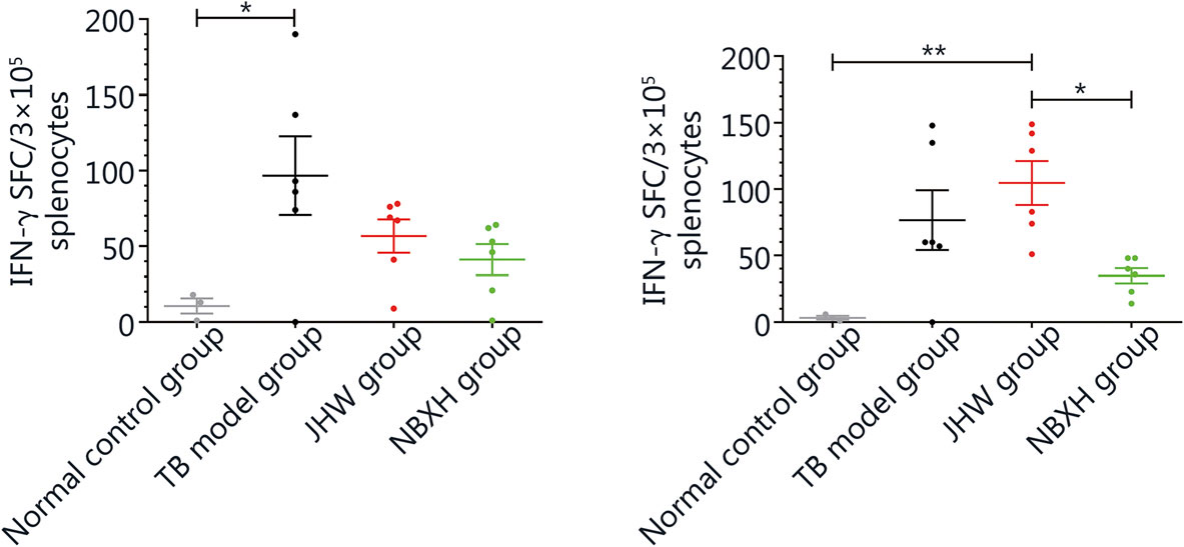
ELISPOT analysis of splenocytes of mice in each group after treatment for 3 weeks (left) and 13 weeks (right). *P < 0.05, **P < 0.01

After 3 weeks of treatment, the numbers of spots representing effector T cells secreting IFN-γ in the TB model group, JHW group, and NBXH group were all higher than that in the normal control group, but only the increase in the TB model group was statistically significant (*P* < 0.05). After 13 weeks of treatment, the numbers of spots for effector T cells secreting IFN-γ in the TB model group, JHW group, and NBXH group were still higher than that in the normal control group, but only the increase in the JHW group was statistically significant, and that in the JHW group was significantly higher than that in the normal control group and NBXH group (*P* < 0.05). Compared with the TB model group, the JHW group and NBXH group did not show significant differences in the number of spots for effector T cells secreting IFN-γ (*P* > 0.05, Fig. 3).

### Expression of 7 inflammatory cytokines

**Fig. 4:**
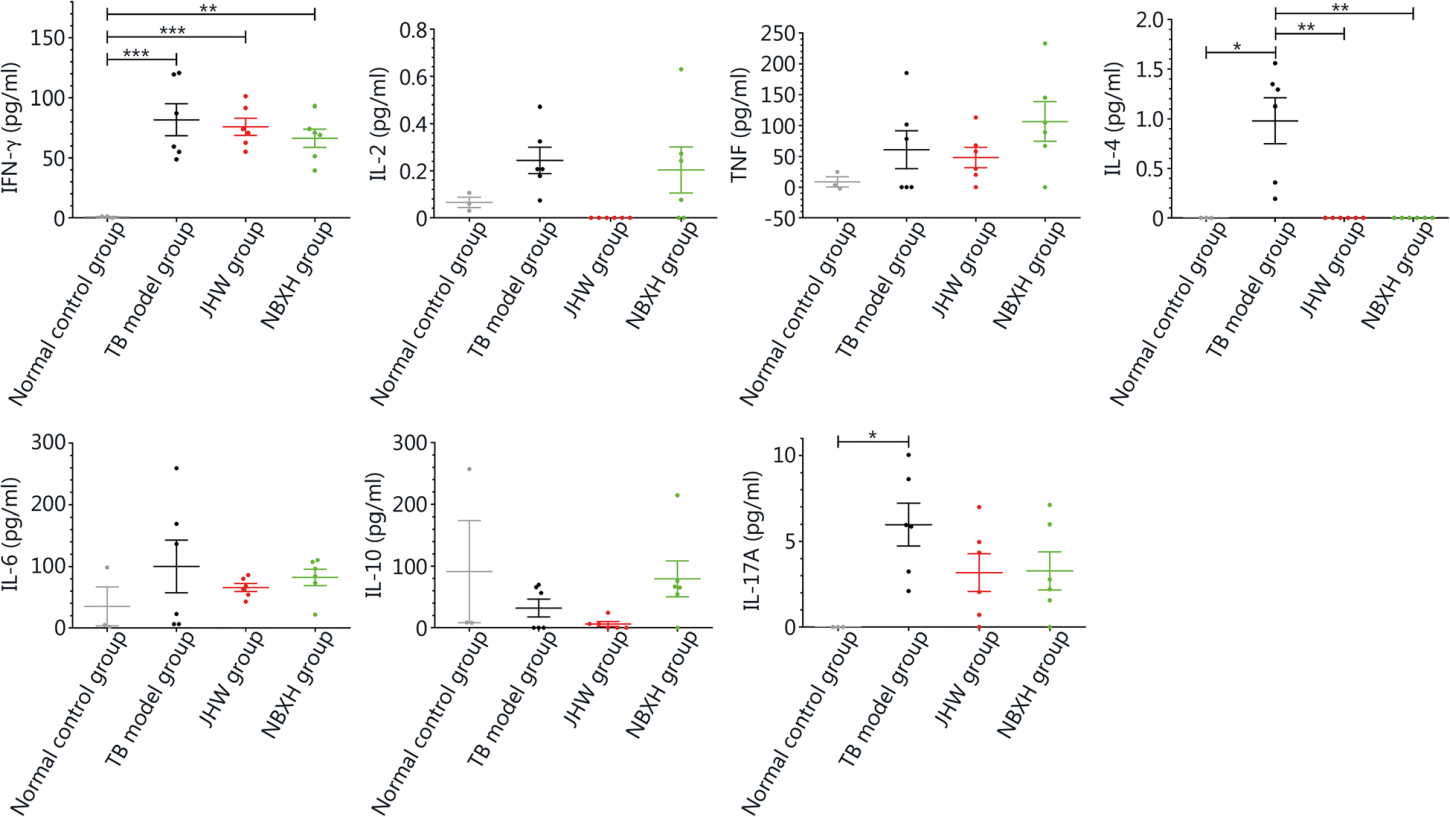
Levels of cytokines in the spleen lymphocyte culture supernatants of mice in each group after treatment for 3 weeks. The cytokines detected were IFN-γ, IL-2, TNF, IL-4, IL-6, IL-10 and IL-17A, respectively. *P < 0.05, **P < 0.01, ***P < 0.001, ^#^*P* < 0.0001

**Fig. 5:**
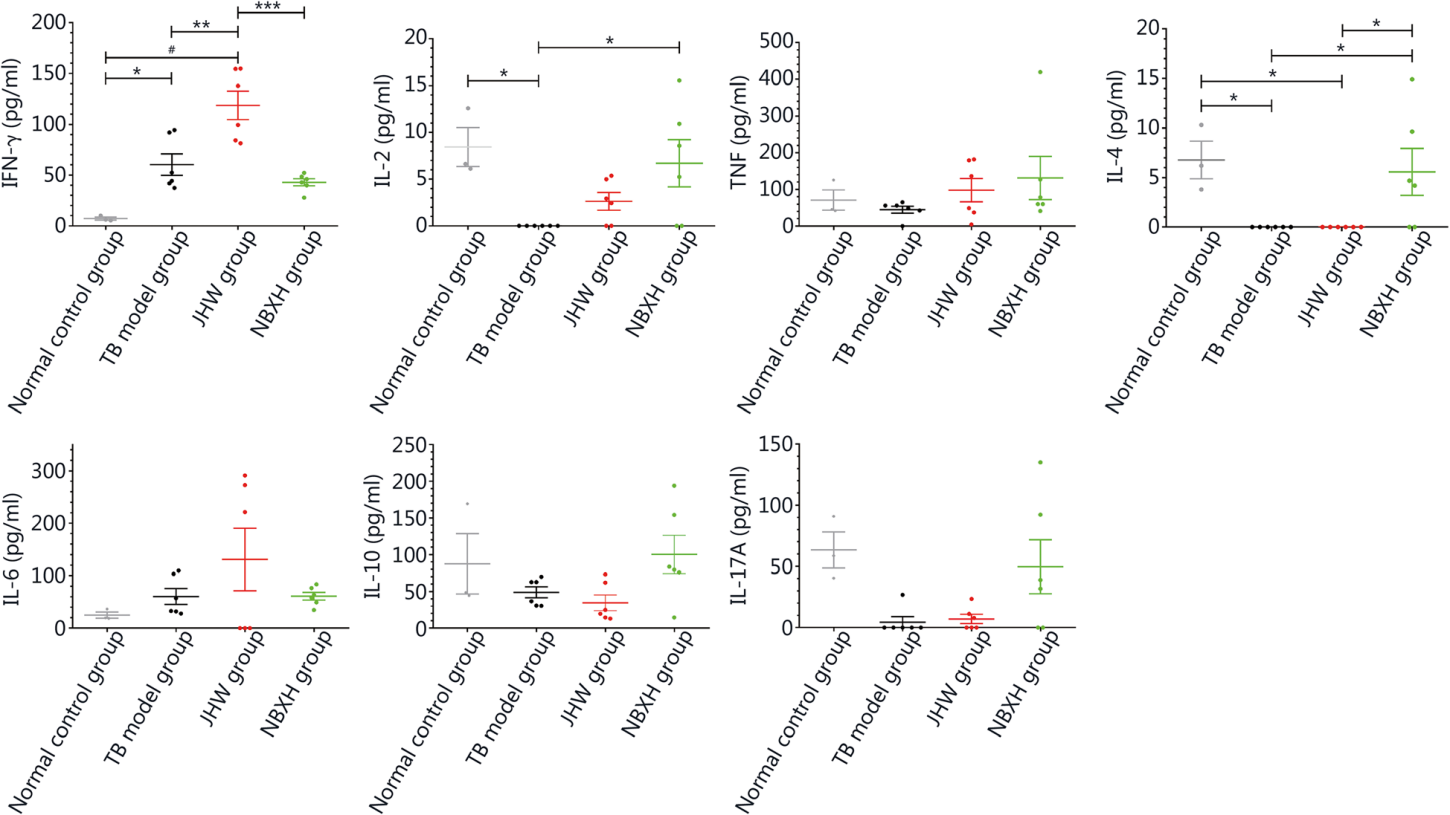
Levels of cytokines in the spleen lymphocyte culture supernatants of mice in each group after treatment for 13 weeks. The cytokines detected were IFN-γ, IL-2, TNF, IL-4, IL-6, IL-10 and IL-17A, respectively. *P < 0.05, **P < 0.01, ***P < 0.001, ^#^*P* < 0.0001

After 3 weeks of treatment, compared with that in the normal control group, the IFN-γ levels in the TB model group, JHW group, and NBXH group were significantly increased (*P* < 0.01), but there were no significant differences among the TB model group, JHW group, and NBXH group (*P* > 0.05). The IL-4 levels in the normal control group, JHW group, and NBXH group were significantly lower than that in the TB model group (*P* < 0.001). The IL-17A level in the TB model group was significantly higher than that in the normal control group (*P* < 0.05), but there were no significant differences among the other groups (*P* > 0.05). There were no significant differences in IL-2, TNF, IL-6, or IL-10 levels (*P* > 0.05, Fig. 4). After 13 weeks of treatment, the IFN-γ levels of the TB model group and the JHW group were still significantly higher than that of the normal control group (*P* < 0.05). Compared with that of the TB model group, the IL-2 levels of the normal control group and NBXH group were significantly increased (*P* < 0.05). There was no significant difference in TNF levels among the groups (*P* > 0.05), but the TNF level in the NBXH group was highest. The IL-4 levels of the normal control group and NBXH group were significantly higher than those of the TB model group and JHW group (*P* < 0.05). There were no significant differences in IL-6, IL-10, or IL-17A levels among the groups (*P* > 0.05), but the IL-17A level was increased in the NBXH group (Fig. 5).

### Identification of DE genes before and after *M. tuberculosis* infection or JHW or NBXH treatment

Heat map and volcano map are used to show the changes of gene expression profiles before and after M. tuberculosis infection, JHW or NBXH treatment (Figs. S1, S2). The results showed that 2774 up-regulated and 2266 down-regulated DE genes were identified in the TB model group compared with the normal control group. A total of 148 up-regulated and 210 down-regulated DE genes were identified in the JHW group compared with the TB model group. A total of 220 up-regulated and 278 down-regulated DE genes were identified in the NBXH group compared with the TB model group. Of the top 30 up-regulated DE genes after NBXH treatment, 19 were down-regulated in the TB model group, and 11 were also up-regulated in the JHW group (shown in Table 1). Of the top 30 down-regulated DE genes after NBXH treatment, 23 were up-regulated in the TB model group, 11 were down-regulated in the JHW group, and 2 were up-regulated in the JHW group (shown in Table 2).

**Table 1:** Top 30 significantly up-regulated DE genes and GO annotations after NBXH treatment

Gene symbol	Fold change in DE			Gene ontology annotations		
	NBXH group vs. TB model group	TB model group vs. normal control group	JHW vs. TB model group	BP	CC	MF
Rho	4.07	NO	NO	NO	NO	NO
Sap25	3.13	NO	NO	NO	NO	NO
Srp54a	2. 50	− 1.16	3.25	SRP-dependent cotranslational protein targeting to membrane; response to drug	Nuclear speck	GTP binding, GTPase activity, drug binding, etc.
Mouse_newGene_24952	2.06	−1.17	2.87	NO	NO	NO
Gm38394	1.89	−1.35	NO	NO	NO	Transcription factor activity, sequence-specific DNA binding, RNA polymerase II regulatory region sequence-specific DNA binding, protein dimerization activity
Mouse_newGene_25840	1.76	−3.91	NO	NO	NO	NO
Mouse_newGene_3409	1.64	−3.60	NO	Humoral immune response mediated by circulating immunoglobulin, positive regulation of peptidyl-tyrosine phosphorylation, early endosome to late endosome transport, etc.	Immunoglobulin complex, circulating, external side of plasma membrane, B cell receptor complex	Antigen binding, transmembrane signaling receptor activity
Mouse_newGene_21033	1.51	−1.37	NO	NO	NO	NO
Mouse_newGene_34719	1.37	NO	NO	NO	NO	GTPase activator activity
Mouse_newGene_1240	1.28	−1.17	1.16	Negative regulation of peptidase activity	Extracellular region	Ligase activity, serine-type endopeptidase inhibitor activity
Mouse_newGene_4366	1.26	NO	1.63	NO	NO	NO
Tmem229a	1.26	NO	NO	NO	NO	NO
Mouse_newGene_3520	1.24	−0.91	0.98	Response to hormone, positive regulation of stress-activated MAPK cascade, negative regulation of microtubule depolymerization, etc.	Cytoplasmic microtubule	Ligase activity, ubiquitin protein ligase binding
Rapgef5	1.23	−1.28	0.92	Small gtpase-mediated signal transduction, positive regulation of gtpase activity		GTP-dependent protein binding
Mapk14	1.22	NO	0.75	Lipopolysaccharide-mediated signaling pathway, response to muscle stretch, etc.	Spindle pole	Protein phosphatase binding, protein c-terminus binding
Slc5a1	1.17	NO	NO	NO	NO	NO
Kcna2	1.16	−2.43	NO	NO	NO	NO
Mouse_newGene_31701	1.14	NO	0.98	Response to oxidative stress, negative regulation of cysteine-type endopeptidase activity involved in apoptotic process, etc.	Lamellipodium, CD95 death-inducing signaling complex, growth cone, etc.	Rac GTPase binding, GTPase activator activity, diacylglycerol choline phosphotransferase activity, etc.
Crispld2	1.13	NO	NO	Lung development, face morphogenesis, extracellular matrix organization	Proteinaceous extracellular matrix, transport vesicle	Heparin binding
Mouse_newGene_25226	1.13	−1.03	NO	NO	NO	NO
Plekhs1	1.11	−0.86	NO	NO	NO	NO
Pcdhgb7	1.11	NO	NO	Nervous system development, synapse organization, cell-cell signaling, etc.	NO	NO
Zfp273	1.10	−1.91	NO	NO	NO	NO
Gpr27	1.09	NO	1.33	Positive regulation of insulin secretion involved in cellular response to glucose stimulus	NO	NO
Mouse_newGene_25949	1.09	−1.81	1.05	Response to lead ion	Growth cone, filopodium	Cholesterol binding
Mouse_newGene_12272	1.09	−1.33	NO	NO	NO	NO
Jmjd8	1.09	−0.88	0.73	NO	NO	NO
Mouse_newGene_6388	1.08	−2.82	NO	NO	NO	NO
Arhgap19	1.07	−1.30	NO	Signal transduction	NO	GTPase activator activity
Zkscan7	1.06	−1.75	NO	NO	NO	NO

The data are displayed as the logarithm of the fold change [log_2_(FC)]. Positive number, up-regulated; Negative number, down-regulated; NO. Not found in the database

*DE* differentially expressed, *GO* gene ontology, *BP* biological process, *CC* cellular component, *MF* molecular function, *NBXH* NiuBeiXiaoHe, *TB* tuberculosis, *JHW* JieHeWan

**Table 2:** Top 30 significantly down-regulated DE genes and GO annotations after NBXH treatment

Gene symbol	Fold change in DE			Gene ontology annotations		
	NBXH group vs. TB model group	TB model group vs. normal control group	JHW vs. TB model group	BP	CC	MF
Zc3h11a	−5.22	2.08	NO	poly(A) + mRNA export from nucleus	NO	NO
C5ar1	−2.98	1.07	NO	Positive regulation of vascular endothelial growth factor production, negative regulation of neuron apoptotic process, positive regulation of macrophage chemotaxis, etc.	Cell surface, apical part of cell, cytoplasmic vesicle	NO
Mouse_newGene_1010	−2.88	NO	NO	NO	NO	NO
Matn4	−2.68	2.41	−3.11	Response to axon injury	Extracellular region	NO
Mouse_newGene_34078	−2.62	1.57	NO	NO	NO	NO
Mouse_newGene_22362	−2.58	NO	−9.68	Translation, ribosomal small subunit assembly, single organismal cell-cell adhesion, etc.	Basement membrane, neuronal cell body, cytosolic small ribosomal subunit, etc.	Structural constituent of ribosome, laminin binding, ribosome binding
Mouse_newGene_31321	−2.54	NO	NO	NO	NO	NO
Mouse_newGene_3400	−2.32	2.25	NO	Mucosal immune response, humoral immune response mediated by circulating immunoglobulin	Immunoglobulin complex, circulating	Antigen binding
Rpl10-ps3	−2.24	2.31	NO	rRNA processing, SRP-dependent cotranslational protein targeting to membrane, liver regeneration, etc.	Cytosolic large ribosomal subunit, polysome	Structural constituent of ribosome
Mouse_newGene_59	−2.23	1.35	−2.01	erythrocyte differentiation	NO	NO
Mouse_newGene_11641	−1.93	4.46	NO	Response to oxidative stress, negative regulation of cell proliferation, negative regulation of mitotic cell cycle, etc.	NO	NO
Mouse_newGene_10816	−1.90	2.61	−1.54	Negative regulation of catalytic activity	NO	Enzyme binding
Mouse_newGene_11438	−1.88	4.921	2.69	Regulation of protein ubiquitination	NO	NO
Mouse_newGene_35672	−1.88	1.31	NO	NO	NO	Ligase activity
Pcdhgc3	−1.86	2.17	NO	Nervous system development, synapse organization, cell-cell signaling, etc.	Cell-cell junction	NO
Mouse_newGene_8457	−1.77	1.18	NO	Lipoprotein metabolic process, lipid transport	Extracellular region	Lipid binding, signal transducer activity
Plcxd1	−1.70	NO	NO	Lipid metabolic process	NO	NO
Mouse_newGene_25732	−1.70	1.77	−0.74	NO	NO	antigen binding
Zc3h11	−1.69	0.94	−0.92	poly(A) + mRNA export from nucleus	NO	NO
Gm29094	−1.65	3.07	NO	Intrinsic apoptotic signaling pathway in response to DNA damage, negative regulation of cysteine-type endopeptidase activity involved in apoptotic process, extrinsic apoptotic signaling pathway in absence of ligand, etc.	Mitochondrial outer membrane	Ligase activity, cysteine-type endopeptidase inhibitor activity involved in apoptotic process
Mouse_newGene_3696	−1.57	1.10	−1.31	Regulation of RNA splicing, mrna processing	Catalytic step 2 spliceosome	Single-stranded RNA binding, TBP-class protein binding, nucleotide binding
Mouse_newGene_21297	−1.56	3.74	−1.38	NO	NO	NO
Stac3	−1.45	NO	NO	Skeletal muscle contraction, skeletal muscle fiber development, neuromuscular synaptic transmission, etc.	NO	NO
Msi1	−1.42	NO	NO	Response to hormone, epithelial cell differentiation	Polysome	Nucleotide binding, poly(u) rna binding
Ccnb1ip1	−1.42	3.06	0.81	Protein ubiquitination, spermatid development	Synaptonemal complex	Ligase activity, ubiquitin protein ligase activity
Mouse_newGene_16290	−1.36	NO	−2.61	Locomotory behavior, nervous system development, social behavior	Dense core granule	Aminoacyl-trna ligase activity
Mouse_newGene_15544	−1.36	1.33	NO	NO	NO	NO
Mouse_newGene_27195	−1.36	1.85	NO	Cellular response to estradiol stimulus, response to nutrient levels, glucocorticoid biosynthetic process	Rough endoplasmic reticulum, apical part of cell, nuclear membrane	11-beta-hydroxysteroid dehydrogenase [nad(p)] activity, steroid binding, nadp binding
Mouse_newGene_5048	−1.31	2.30	−0.85	NO	NO	NO
Mouse_newGene_3462	−1.31	1.56	−1.36	NO	NO	NO

The data are displayed as the logarithm of the fold change [log_2_(FC)]. Positive number, up-regulated; Negative number, down-regulated; NO. Not found in the database

*DE* differentially expressed, *GO* gene ontology, *BP* biological process, *CC* cellular component, *MF* molecular function, *NBXH* NiuBeiXiaoHe, *TB* tuberculosis, *JHW* JieHeWan

### GO analyses of DE genes before and after *M. tuberculosis* infection or JHW or NBXH treatment

**Fig. 6:**
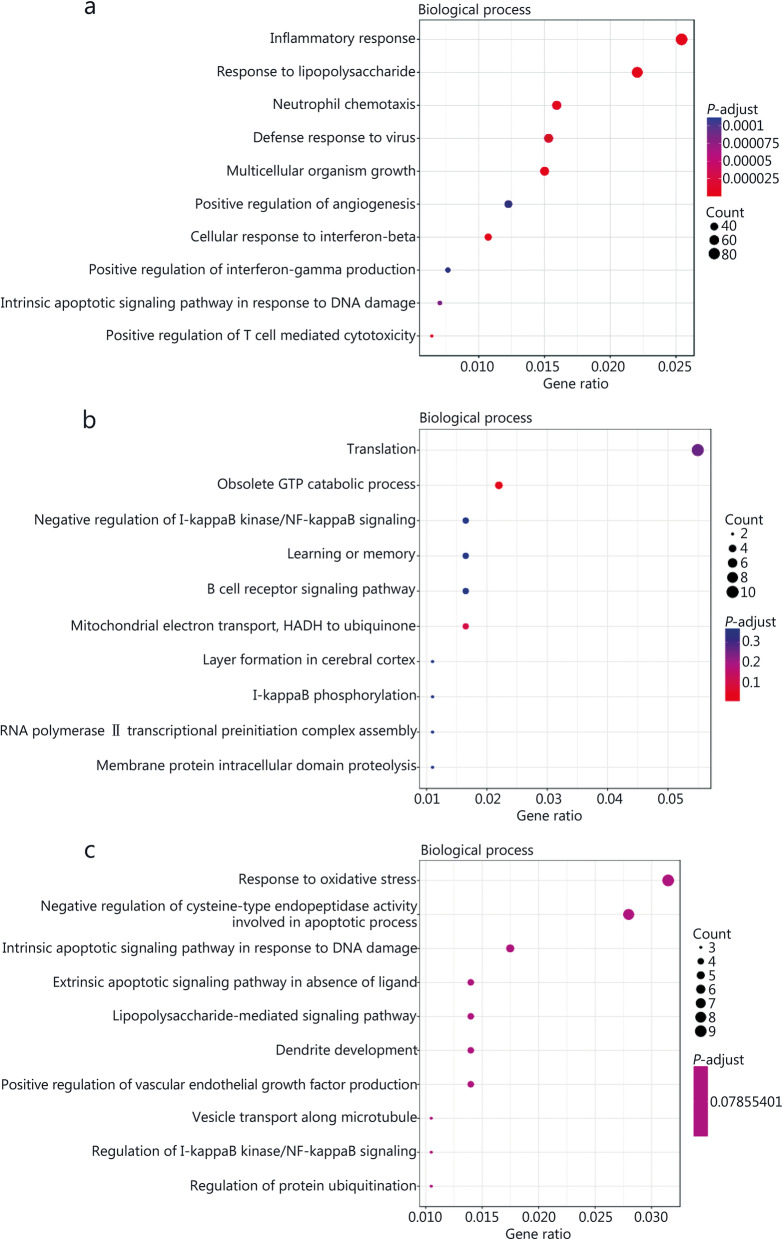
Gene ontology (GO) analyses of significant differentially expressed (DE) genes identified by comparing before and after *M. tuberculosis* infection or NiuBeiXiaoHe (NBXH) treatment in biological process. The abscissa is the gene ratio, which is the ratio of the gene of interest annotated in the term to the number of all DE genes, and the ordinate is every GO term. Dot size represents the number of DE genes annotated in the pathway, and dot color represents the corrected *P*-value of the hypergeometric test. **a** Tuberculosis (TB) model group vs. normal control group. **b** JieHeWan (JHW) vs. TB model group. **c** NBXH vs. TB model group.

**Fig. 7:**
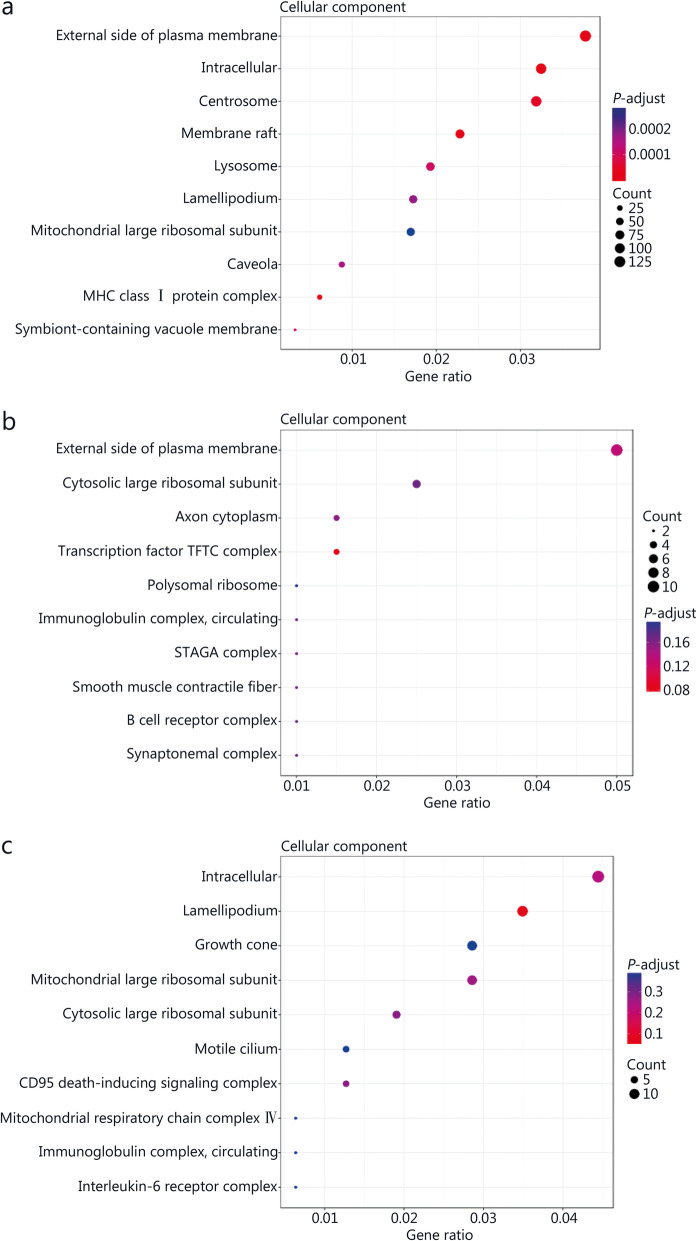
Gene ontology (GO) analyses of significant differentially expressed (DE) genes identified by comparing before and after *M. tuberculosis* infection or NiuBeiXiaoHe (NBXH) treatment in cellular component. The abscissa is the gene ratio, which is the ratio of the gene of interest annotated in the term to the number of all DE genes, and the ordinate is every GO term. Dot size represents the number of DE genes annotated in the pathway, and dot color represents the corrected *P*-value of the hypergeometric test. **a** Tuberculosis (TB) model group vs. normal control group. **b** JieHeWan (JHW) vs. TB model group. **c** NBXH vs. TB model group.

**Fig. 8:**
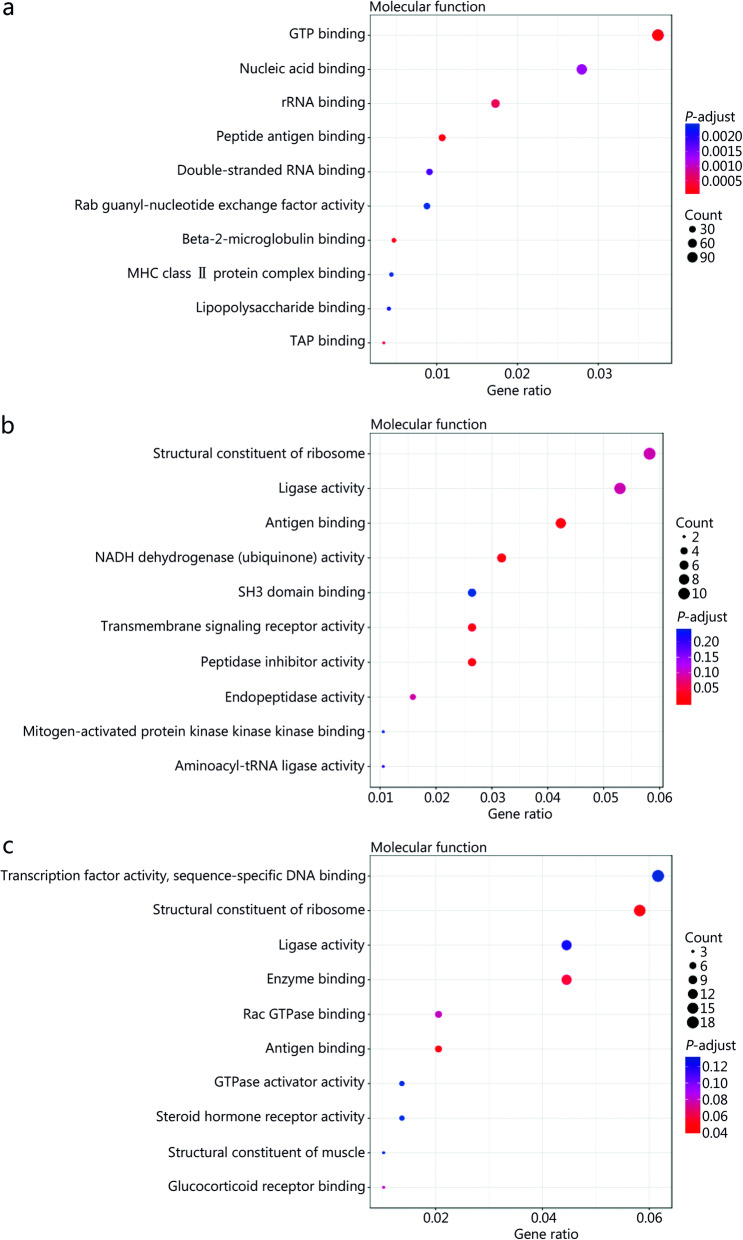
Gene ontology (GO) analyses of significant differentially expressed (DE) genes identified by comparing before and after *M. tuberculosis* infection or NiuBeiXiaoHe (NBXH) treatment in molecular function. The abscissa is the gene ratio, which is the ratio of the gene of interest annotated in the term to the number of all DE genes, and the ordinate is every GO term. Dot size represents the number of DE genes annotated in the pathway, and dot color represents the corrected *P*-value of the hypergeometric test. **a** Tuberculosis (TB) model group vs. normal control group. **b** JieHeWan (JHW) vs. TB model group. **c** NBXH vs. TB model group

The top 10 biological processes (BP) (Fig. 6), cellular components (CC) (Fig. 7), and molecular function (MF) (Fig. 8) GO terms with statistical significance for DE genes identified after *M. tuberculosis* infection or traditional Chinese medicines treatment are shown in Figs. 6, 7 and 8.

### Pathway analyses of DE genes before and after *M. tuberculosis* infection or JHW or NBXH treatment

**Fig. 9:**
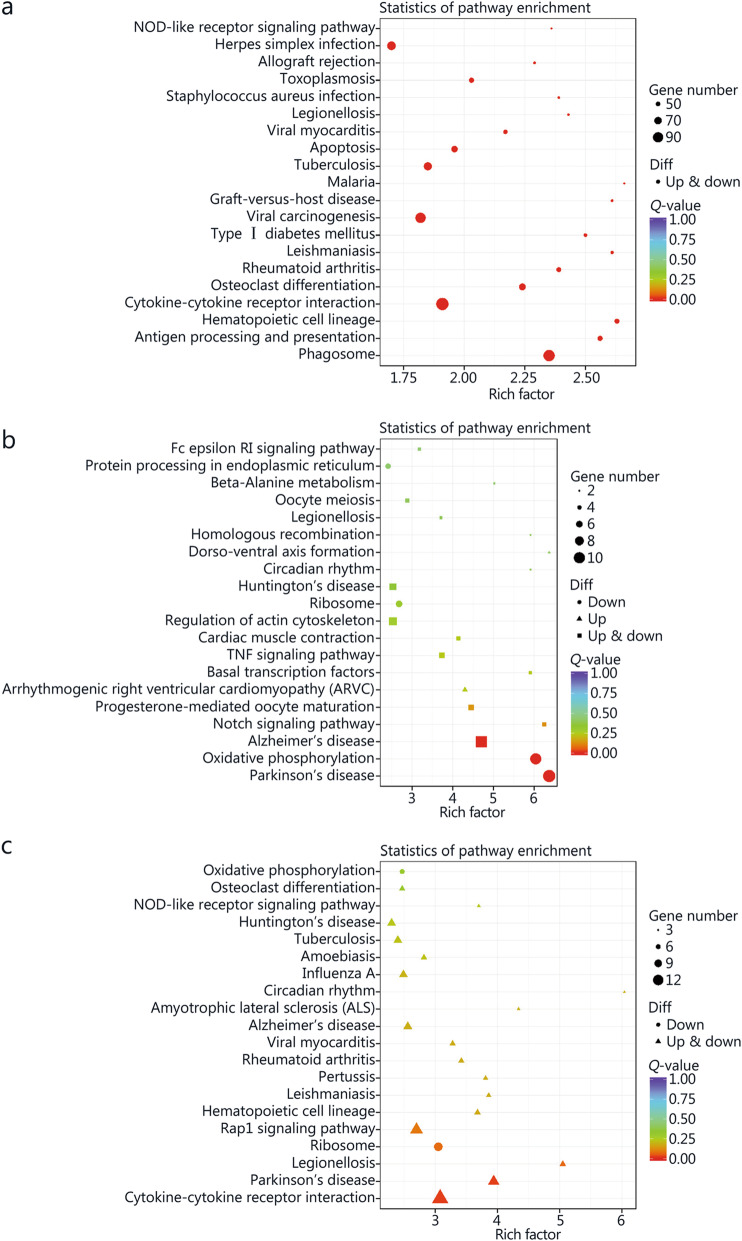
Kyoto Encyclopedia of Genes and Genomes (KEGG) analyses of significant differentially expressed (DE) genes identified by comparing before and after *M. tuberculosis* infection or NiuBeiXiaoHe (NBXH) treatment. The abscissa is the enrichment factor, indicating the ratio of the gene proportion annotated to the pathway in the DE genes to that annotated to the pathway in all genes. The larger the enrichment factor, the more significant the enrichment level of DE genes in this pathway. **a** Tuberculosis (TB) model group vs. normal control group. **b** JieHeWan (JHW) vs. TB model group. **c** NBXH vs. TB model group

The molecular pathways of the identified DE genes were annotated by KEGG analysis. The results for the top 20 pathways closely related to the disease pathogenesis are shown in Fig. 9.

## Discussion

The treatment of TB with a combination of traditional Chinese medicine and chemotherapeutic drugs can achieve a comprehensive therapeutic effect, reduce the symptoms of TB, reduce the liver and kidney damage induced by chemotherapeutic drugs, reduce complications, and improve the treatment effect in TB patients [10]. According to the report by Yang QS et al. [11], JHW has anti-*M. tuberculosis* activity, can enhance host immune function and has a certain therapeutic effect on many kinds of TB, such as treatment-naive and previously treated pulmonary TB, drug-resistant TB, and senile TB. Comparing the anti-TB effects of NBXH and JHW in vivo will provide a reliable theoretical basis for the clinical application of the anti-TB Chinese medicine compound NBXH.

The results of previous studies by our team showed that NBXH has a distinct degree of therapeutic effect on the mouse TB model, improving the general condition, reducing organ colony counts, and decreasing the severity of TB lesions [12]. To better understand the difference in the anti-TB effect between NBXH and traditional Chinese medicine in clinical application, this study compared the anti-TB effect of an NBXH powder newly produced by Xi’an XinTong Pharmaceutical Research Co., Ltd. and that of JHW in vivo. In this study, the TB model was confirmed to be successfully established by observing the lung tissue pathology and organ colony counts of mice infected with *M. tuberculosis* for 3 days.

The pathogenic mechanism of *M. tuberculosis* infection mainly involves the growth and metabolism of *M. tuberculosis* inducing the host immune system to produce an inflammatory response and cause immune damage. After *M. tuberculosis* infects the body, different types of pathological changes, such as exudation, hyperplasia, and necrosis, may occur depending on the number of infectious bacteria, bacterial virulence, host immune response, and tissue characteristics [13, 14]. The results published by Xiang ZG et al. [15] showed that 1 week after acute *M. tuberculosis* infection in mice, inflammatory cell exudation and interstitial edema appeared in the lungs, and inflammatory cell aggregation occurred in the spleen, 3 weeks later, granulomas appeared in the lungs and spleen, and the area and quantity of the inflammatory reaction increased with the extension of infection time. The application of HuangKuiSu could significantly reduce the pathological damage in lung tissue in mice infected with *M. tuberculosis* and reduce lung colony counts [16]. The application of FeiLaoKang could also significantly inhibit the inflammatory response in the lungs in a mouse TB model and significantly improved inflammatory symptoms [17]. The results of this study are consistent with these literature reports. The pathological examination of lung tissue in this study showed that the pathological changes in the NBXH group and JHW group were significantly less severe than those in the TB model group after 3 weeks of treatment, and the pathological area in the NBXH group was smaller than those in the TB model group and JHW group after 13 weeks of treatment. This shows that NBXH induces a better anti-TB inflammatory response and reduces pathological damage.

Organ bacterial loads are used to evaluate the antibacterial or bactericidal effect of anti-TB drugs in vivo. Organ bacterial loads correspond to the infection dose and last for at least 14 weeks [18]. The lower the organ bacterial loads are, the stronger the bactericidal effect of drugs, and the better the therapeutic effect [19]. Jiang JQ et al. showed that traditional Chinese medicine also had a certain bacteriostatic or bactericidal effect on *M. tuberculosis*, such as high-dose JHW significantly reducing lung and liver colony counts in guinea pigs (compared with the blank control group, the treated group showed a reduction of 272 CFUs/10 mg in the lungs and a reduction of 181 CFUs/10 mg in the liver), and reduces lung tissue lesions [20]. Our study showed that there were no significant differences in the bacterial loads of the lungs or liver in each group after 3 weeks of treatment, indicating that the treatment time of traditional Chinese medicines were too short to have a significant bacteriostatic or bactericidal effect. In contrast, after 13 weeks of treatment, the bacterial loads of the lungs in the NBXH group and JHW group were significantly lower than those in the TB model group, indicating that long-term traditional Chinese medicine treatment may have better bacteriostatic and bactericidal effects on the lungs.

Traditional Chinese medicine can exert an anti-TB effect by regulating the host immune system. Helper T lymphocyte subsets and cytokines secreted by these cells play an important role in the process of anti-TB immunity. The Th1-type immune response is key in the anti-*M. tuberculosis* infection response. IFN-γ, IL-2, and TNF are the key immune indexes in the Th1-type immune response. The expression of IFN-γ plays an important role in the anti-TB immune response, and the expression level in the body is closely related to TB [21–23]. IFN-γ can promote the proliferation and differentiation of T cells, activate macrophages, and enhance the phagocytic activity and killing effect of T cells and macrophages on *M. tuberculosis*. IL-2 is an important factor in regulating the immune response, promoting the production of IFN-γ, and inducing the differentiation of killer cells, such as natural killer cells. TNF can cooperate with IFN-γ to enhance the expression of inducible nitric oxide synthetase and the antibacterial effect of macrophages and promote the formation of tuberculous granuloma [24, 25]. After infection with *M. tuberculosis*, the expression level of IFN-γ generally increases, and after 2 months of intensive anti-TB treatment, the level of IFN-γ decreases in responsive patients [26]. When the secretion of IL-2 and TNF is insufficient, the immune response cannot completely eliminate *M. tuberculosis* but is increased with the improvement in TB after anti-TB treatment [27]. The cytokines secreted by Th2 cells are mainly IL-4, IL-6, and IL-10, and their main functions are to promote the development of B cells and induce a humoral immune response. IL-4 mainly promotes the differentiation of Th0 cells into Th2 cells and inhibits the expression of cytokines related to Th1 cells [28]. IL-6 is a B cell stimulator that can induce B cells to undergo differentiation and maturation to produce antibodies and participate in the anti-inflammatory effect of the body, at the same time, IL-6 can cooperate with colony-stimulating factor (CSF) to promote the growth and differentiation of primitive bone marrow-derived cells and enhance the lytic function of natural killer (NK) cells. Liu WS et al. showed that the higher the level of IL-6 is in vivo, the more severe the lung inflammation [29]. IL-10 is an anti-inflammatory cytokine that can reduce the overactivation of coactivators in the phagocyte system to weaken antigen presentation [30]. It has been reported that the long-term control of TB infection requires not only the enhancement of the Th1-type immune response but also the inhibition of the Th2-type immune response [31]. Lv Y et al. [32] showed that the mice infected with attenuated *M. tuberculosis* strain H37Ra mainly produced a Th1-type immune response. IL-17A is an inflammatory factor mainly produced by activated T cells that is induced in the early stage of *M. tuberculosis* infection and can promote the activation of T cells, participate in the recruitment of neutrophils and induce various cytokines, such as IL-6, IL-8, and granulocyte-macrophage colony-stimulating factor (GM-CSF) [33–35]. In addition, IL-17A participates in the formation of mature granuloma in the lungs during *M. tuberculosis* infection, which plays a key role in the prevention of *M. tuberculosis* spread [36]. In TB patients, Th1-type cytokines provide protective immunity, and Th2-type cytokines may promote the inflammatory response and immune damage, while Th17 cytokines play roles in both protection and pathological damage, regulating the T cell response is essential for promoting anti-*M. tuberculosis* immunity and preventing widespread immune pathology [37, 38]. In this study, results for the number of spots representing effector T cells secreting IFN-γ and the levels of cytokines in the culture supernatant of splenic lymphocytes showed that after 3 weeks of treatment, the IFN-γ levels in the TB model and two Chinese medicine treatment groups were significantly increased and the IL-4 level in the TB model group was also significantly increased, while the level of Th2-type cytokines did not increase in the two traditional Chinese medicine treatment groups, which suggested that Th1 and Th2 immune response were both enhanced in the TB model group, while the Th1 immune response was dominant in the two traditional Chinese medicine treatment groups. After 13 weeks of treatment, the IFN-γ level in the TB model group and especially in the JHW group was still significantly increased, but the Th2 cytokine levels were not significantly increased, which indicated that the Th1 immune response was the main response and the Th2 immune response was inhibited in these two groups. The cytokine profile of the NBXH group changed, the IFN-γ level decreased, the IL-2 level significantly increased, and the IL-4 and IL-17A levels increased significantly, which suggested that the Th1 immune response and Th2 immune response tended to be balanced. To date, studies have shown that in TB patients given effective anti-TB treatment, IFN-γ levels decrease while IL-2 levels increase [26, 27], and these changes in the cytokine profile suggest effective NBXH treatment. In addition, some reports [39, 40] have shown that the recovery of Th1 and Th2 immune response balance in the treatment of TB is beneficial for the prognosis of patients. NBXH may play a role by regulating the Th1/Th2 immune balance rather than simply enhancing the Th1 response or weakening the Th2 response. After 13 weeks of treatment, the increase in the IL-17A level in some mice was conducive to the formation of granulomas and limited the spread of *M. tuberculosis*, which may also be one of the reasons for the reduced pathological damage in the NBXH group.

By comparing the changes in the gene expression profile before and after treatment with traditional Chinese medicine, we can understand the target of traditional Chinese medicine, clarify the mechanism of traditional Chinese medicine, and establish a new way to study the modernization of traditional Chinese medicine. At present, some research reports [41] have performed beneficial explorations, but we are the first to use transcriptome sequencing technology to analyze the abundance of mouse cell gene transcripts to evaluate the effect of anti-TB Chinese medicine treatment and explore the molecular mechanism of the effect. This study indirectly reflected the difference in gene expression by comparing the difference in gene transcript abundance between the TB model group and the normal control group and found 2774 up-regulated and 2266 down-regulated DE genes, which is consistent with previous research reports [42, 43], which found that mycobacterial infection can cause significant changes in the gene expression profile of macrophages. Through GO and KEGG analyses, it was found that the cell damage that occurred after *M. tuberculosis* infection was relatively extensive, showing significant changes in terms for cell components, molecular functions and biological processes, the enriched GO terms related to BP were mainly inflammatory response, response to lipopolysaccharide, neutrophil chemotaxis, cellular response to interferon-beta, positive regulation of interferon-gamma production, intrinsic apoptotic signaling pathway in response, and positive regulation of T cell-mediated cytotoxicity, indicating that *M. tuberculosis* infection has a significant effect on the host immune response and inflammatory response. KEGG pathway analysis also shown that the pathways involved in antigen recognition, phagocytosis, apoptosis, antigen processing, and presentation by innate immune cells to adaptive immune cells all showed changes in up- or down-regulated pathways, such as the NOD-like receptor signaling pathway, phagosome, apoptosis, antigen processing and presentation, and cytokine-cytokine receptor interaction. On the one hand, these findings support the current understanding of the pathogenesis of TB, that is, TB may be caused by inflammation and immune damage caused by *M. tuberculosis* proliferation in tissue cells [44]. On the other hand, they are helpful to further clarify the pathogenic mechanism of *M. tuberculosis*.

In this study, 148 up-regulated and 210 down-regulated DE genes were identified by comparing the JHW group and the TB model group, and 220 up-regulated and 278 down-regulated DE genes were identified by comparing the NBXH group with the TB model group. These results showed that the number of DE genes after NBXH treatment was slightly higher than that after JHW treatment, which reflects that NBXH has more targets than JHW, and traditional Chinese medicine compound preparations with complex chemical components have multicomponent, multipathway and multitarget therapeutic characteristics. The changes in BP, CC, and MF terms and in signaling pathways caused by treatment with NBXH or JHW were not completely consistent. NBXH treatment had a greater effect on BP terms and signaling pathways related to the immune response and inflammatory response, such as GO terms related to the biological processes response to oxidative stress, intrinsic apoptotic signaling pathway in response, lipopolysaccharide-mediated signaling pathway, regulation of l-kappaB kinase/NF-kappaB signaling, and regulation of protein ubiquitination. KEGG pathway analysis related to signaling pathways included down-regulated oxidative phosphorylation, up- or down-regulated Rap1 signaling pathway, and cytokine-cytokine receptor interaction. Among the pathways closely related to disease, the TB pathway ranked fifth. After JHW treatment, the pathways related to the regulation of the immune and inflammatory response were down-regulated Fc epsilon RI signaling pathway, protein processing in the endoplasmic reticulum and ribosome, up- or down-regulated TNF signaling pathway and notch signaling pathway, and down-regulated oxidative phosphorylation. These results indicate that NBXH treatment mainly plays an anti-TB role by regulating the host immune response and inflammatory response, and its mechanism of action is different from that of JHW, NBXH is more targeted for TB and may be more effective in restoring immune damage caused by TB. The results in Table 1 and Table 2 also support this view. Most of the top 30 significantly up- or down-regulated DE genes were recovered by NBXH treatment after *M. tuberculosis* infection, which is consistent with only approximately one-third of the significantly up- or down-regulated DE genes being restored after JHW treatment, indicating that NBXH treatment has a more significant reparative effect on cell damage caused by *M. tuberculosis* infection, which may be due to the different components, targets and mechanisms of action of these two kinds of traditional Chinese medicine prescriptions. In addition, at present, it is difficult to evaluate the effectiveness of traditional Chinese medicine in animal experiments. The theoretical systems of traditional Chinese medicine and Western medicine are totally different. It is usually difficult to obtain significant differences in the evaluation of traditional Chinese medicine by the efficacy evaluation indexes used in Western medicine. The cell damage repair function of NBXH identified by the gene expression profile may also be used as one of the indicators for efficacy evaluation of traditional Chinese medicine. This is because the recovery of significant changes in gene expression caused by pathogenic bacteria will inevitably result in a good change in the phenotype of host cells, such as the pathological damage to lung tissue in the NBXH group being relatively light. In the future, we will further study the efficacy evaluation and modernization of traditional Chinese medicine to provide ideas and experience.

The mechanism of action of NBXH is relatively complicated, involving changes in the expression of many different functional genes. The genes listed in Tables 1 and 2 may be the targets of NBXH and may also be important links in its signal transduction pathway. We analyzed the changes and functions of several representative DE genes to partially clarify the mechanism of action of NBXH. (1) The genes acting on signaling pathways and regulation include the Rho gene, which is a member of the group of guanosine triphosphate (GTP)-binding proteins called Rho GTPases because of their GTPase activity. Rho is a subfamily member of a small G protein superfamily and a member of the Ras superfamily, functions as a key molecule in multiple signal transduction pathways in cells and plays a key regulatory role in a series of cell processes. NBXH-mediated upregulation may play an important role in anti-TB activity in the immune response by affecting the biological processes of activation, proliferation, adhesion, and migration of immune cells such as T lymphocytes, B lymphocytes, monocytes, and macrophages [45, 46]. Rap guanine nucleotide exchange factor 5 (Rapgef5) plays an important role in the process of multicellular differentiation by regulating the Wnt signaling pathway [47]. Gm38394 has transcription factor activity and can control the transcription and expression of DNA by binding with specific DNA sequences, and NBXH can recover and increase its expression to improve the activity of cells. (2) The genes regulating the immune response and inflammatory response include the Mapk14 gene, which is a member of the mitogen-activated protein kinase (MAPK) family. Its functions include extracellular domain activity and protein binding, regulating cell growth, differentiation, oxidative stress, the inflammatory response, and other important cellular physiological/pathological processes [48, 49]. NBXH upregulates Mapk14 expression, regulates the immune response during *M. tuberculosis* infection, induces BAX translocation and apoptosis, enhances TNF expression, limits *M. tuberculosis*, and exerts an anti-TB effect [50]. The protein Crispld2 can bind to LPS, and LPS binds to TLR4 receptors, thereby exerting a feedback regulation mechanism, and its up-regulated expression can reduce the release of inflammatory mediators (such as TNF-α and IL-6), systemic inflammation, and the degree of lung injury [51]. ZC3H11A is a stress-induced protein. Currently, some viruses (such as adenovirus, influenza virus, HIV, and herpes simplex virus) are known to rely on the expression of this gene. NBXH treatment can significantly reduce the expression of this gene [52]. On the one hand, ZC3H11A plays an anti-inflammatory role and may protect cells from damage. On the other hand, it may also contribute to antiviral infection in the body. C5aR1 is the receptor of the complement factor C5a. C5a regulates various biological processes, such as immune inflammation, opsonization, and cell lysis, through the C5aR1 pathway [53]. NBXH significantly downregulates the expression of this gene and may also protect cells from inflammatory damage. (3) The genes regulating cell differentiation and proliferation include the Srp54a gene, which is a 54-kDa signal recognition particle [54] with GTP binding and catalytic activity that mediates protein recognition and transport and participates in the directional transfer of intracellular proteins. NBXH-mediated restoration of the genes down-regulated by *M. tuberculosis* infection plays an important role in cell differentiation and metabolism. (4) The genes regulating energy metabolism include the Slc5a1 gene, which is a glucose transporter [55]. NBXH increases Slc5a1 expression to promote maximal glucose influx into cells to generate energy and promote cell metabolism. Additionally, the anti-TB effect of many newly identified up- and down-regulated genes, as well as the correlations between genes and between genes and proteins, have not been elucidated. Further research is needed to reveal more mechanisms of action and pathways.

## Conclusions

In summary, NBXH had similar therapeutic effects as the patented Chinese drug preparation JHW, which is used in the clinic, in improving lung histopathology, reducing lung colony counts, and regulating the levels of cytokines. NBXH restored significant changes in gene expression caused by *M. tuberculosis* infection by regulating the expression of immune-related genes and activity of signaling pathways and repairing cell damage caused by *M. tuberculosis* infection. In this study, using a gene expression profile to observe the repair of injury caused by *M. tuberculosis* infection after drug treatment is proposed for the first time as an indicator for judging the efficacy of new drugs, which provides the experimental basis for the further development and clinical application of the compound NBXH. We will also study the combined effect of traditional Chinese medicine and chemical medicine to lay the experimental foundation for the establishment of a combined treatment regimen in the future.

## Supplementary Information

**Additional file 1: Fig. S1** Heatmap of the differential expression profile of genes compared between the Tuberculosis (TB) model group and normal control group, JieHeWan (JHW) group or NiuBeiXiaoHe (NBXH) group. The abscissa represents the sample name and sample clustering result, and the ordinate represents the differential gene and gene clustering result. Different columns represent different samples, and different rows represent different genes. The color represents the gene expression level (log_10_) in the sample. The green dots represent down-regulated DE genes, the red dots represent up-regulated DE genes, and the black dots represent nondifferentially expressed genes. **a** TB model group (T01,T02,T03) vs. normal control group (T13,T14,T15). **b** JHW (T04,T05,T06) vs. TB model group. **c** NBXH (T07,T08,T09) vs. TB model group.

**Additional file 2: Fig. S2** Volcano plot of the differential expression profile of genes compared between the Tuberculosis (TB) model group and normal control group, JieHeWan (JHW) group or NiuBeiXiaoHe (NBXH) group. Each point in the differential expression volcano plot represents a gene, the abscissa represents the logarithm of the difference in the expression level of a gene between two samples, and the ordinate represents the negative logarithm of the *P*-value. The green dots represent down-regulated DE genes, the red dots represent up-regulated DE genes, and the black dots represent nondifferentially expressed genes. **a** TB model group vs. normal control group. **b** JHW vs. TB model group. **c** NBXH vs. TB model group.

## Data Availability

The datasets used during the current study are available from the corresponding author upon reasonable request.

## Abbreviations

BP: biological process
CBA: cytometric bead array
CC: cellular component
CFUs: colony-forming units
CSF: colony-stimulating factor
DE: differentially expressed
GM-CSF: granulocyte-macrophage colony-stimulating factor
GO: gene ontology
IFN-γ: interferon gamma
JHW: JieHeWan
IL: interleukin
KEGG: Kyoto Encyclopedia of Genes and Genomes
*M. tuberculosis*: *Mycobacterium tuberculosis*
MDR-TB: multidrug-resistant tuberculosis
MF: molecular function
NBXH: NiuBeiXiaoHe
NK: natural killer
TB: tuberculosis
Th-1: helper T lymphocytes-1
Th-2: helper T lymphocytes-2
TNF: tumor necrosis factor
